# Political Protest in Times of Crisis. Construction of New Frames of Diagnosis and Emotional Climate

**DOI:** 10.3389/fpsyg.2017.01568

**Published:** 2017-09-12

**Authors:** José-Manuel Sabucedo, Idaly Barreto, Gloria Seoane, Mónica Alzate, Cristina Gómez-Román, Xiana Vilas

**Affiliations:** ^1^Psicología Social, Básica y Metodología, Universidade de Santiago de Compostela Santiago de Compostela, Spain; ^2^Faculty of Psychology, Catholic University of Colombia Bogotá, Colombia; ^3^Laboratorio de Innovación Social, Instituto Tecnológico Metropolitano Medellín, Colombia

**Keywords:** frame diagnosis, perception of injustice, emotional climate, protests, context

## Abstract

In times of crisis, political mobilizations increase. Many of them compete to impose a determined diagnosis of the situation. This work analyses this issue, taking into consideration two of the movements that have had a greater incidence during the crisis in Spain: The Catalonian National Assembly and the Marches for dignity. The objective is to know how the categories of aggrieved ingroup and outgroup responsible were identified and how both these movements defined the emotional climate at that moment. This work includes two studies. In the first one, an analysis of the categories identified in the manifestos published by these two movements was carried out. The results show that the Marches for dignity constructed a more inclusive ingroup identity and show a more negative emotional climate than the Catalonian National Assembly. The second study includes a sample of 919 participants and non-participants in 2 demonstrations called by those organizations. In this case MANOVAs of 2 (Type of demonstration: Catalonian National Assembly, Marches for dignity) × 2 (Type of participants: participants, non-participants) were performed. Results show that participants in both demonstrations have a higher level of injustice than non-demonstrators. Furthermore, demonstrators in Marches for dignity have a more negative perception of emotional climate than non-demonstrators. However, and contrary to the hypothesis, demonstrators of the Catalonian National Assembly have a more positive perception of emotional climate than non-demonstrators. The work explains these results in the socio-political context in which each of these movements acts and highlights the relevance of comparative investigation designs to further the knowledge of political mobilization dynamics.

## Introduction

Injustice and grievances are a reality in all periods and societies. However, these adverse situations lead to protests on few occasions. For them to take place, there must firstly be a demand from the citizens. Secondly, groups or movements must create the offer of mobilization (Klandermans, 2004; van Stekelenburg and Klandermans, 2014) based on different analyses of the situation.

In times of crisis, the possibilities of citizen mobilization increase, giving way to what Tarrow (1991) called protest cycles. Tarrow maintains that “although protest waves do not have a regular frequency or extend uniformly to entire populations, a number of features have characterized such waves in recent history” (Tarrow, 1993, p. 284). These ‘features of cyclicity’ are characterized by, among others, a heightened conflict, broad sectoral and geographic extension, the appearance of new social movement organizations and the empowerment of old ones, as well as the creation of new “master frames” of meaning.

The economic and political crisis which started in 2008 was associated with a significant increase in protest action. In the case of Spain, it went from 10,000 demonstrations in 2007 to 15,000 in 2008 and rose to over 40,000 in 2013 (Ministry for Home Affairs of the Spanish Government, 2014). Moreover, and according to cycles of protest, it led to new movements, such as the Indignados and those derived from it as the Marches for dignity, and revitalized older ones, such as the Catalonian Movement for independence. The Marches for dignity demanded change in the Spanish political and economic situation, while the Catalonian movement claimed that Catalonia, a Spanish region, be independent from Spain. Although these movements arise at the same time and as a response to the same situation, they compete to channel the population’s disaffection to reach different objectives. Thus, they need to elaborate persuasive arguments on the causes of this injustice as best they can (Eyerman and Jamison, 1991; Sabucedo et al., 1998).

### Frame of Diagnosis and Emotional Climate

The messages movements use are what Goffman (1974) called frames, cognitive structures which guide the perception and interpretation of reality. Gamson (1992) used the expression frames of collective action to refer to the beliefs that certain groups spread to legitimize and justify the protest. One of these frames is the diagnosis of the situation. This is especially relevant because it refers to two tightly related and basic questions for mobilization: the definition of a situation as unfair (Bergstrand, 2014) and the construction of an aggrieved identity (Simon and Klandermans, 2001).

According to this, and considering that people belong to different social categories: sex, age, ethnic group, community/country, profession, etc., the perception of injustice may be associated to belonging to any of those groups. When there is a political and economic crisis that affects the majority of the population, the alternative is to construct an identity as inclusive as possible or to locate oneself in one of the other, more exclusive, identities (Sabucedo et al., 2010). This means that in the same situation of injustice, different group identities can be constructed and identify the people and/or organizations responsible. According to Cristancho (2015), identifying who is blamed for the political and economic crisis is a central indicator of how the crisis is perceived.

Apart from these cognitive elements, the frames also include an emotional dimension, as is set out in the intergroup emotions theory (IET) (Smith, 1993). The IET assumes two important theoretical approaches in social psychology. On one hand, the social identity theory and self-categorization theory (Turner and Killian, 1987), which point out that the higher the level of identification of the individual with the group, the more probable a response in group terms will be. On the other hand, the cognitive appraisal theory posits that emotions are the response to a determined evaluation of the setting. This means the diagnosis frame lets people assess their setting and the resources they possess to deal with it (Lazarus, 1984; Frijda, 1988). Emotions are thus going to depend on the framing which individuals give to what happens around them.

One of these possible frames, is the one raised by Gamson (1992), who stated that the perception of injustice is associated with anger. This emotion plays an important motivating role insofar as it “puts fire in the belly and iron in the soul” (Gamson, 1992, p. 32). The direct or indirect influence of anger on participation in political protest has wide empirical support (van Zomeren et al., 2008; van Stekelenburg et al., 2011).

Although anger has been the most negative emotion studied in collective action, other equally important ones exist to understand behavior associated with certain adverse situations. This is the case of fear or sadness. The former is a response to a social environment perceived as threatening and it manifests in escape or protection; and sadness is a consequence of loss caused by the outgroup and leads to the suspension of any kind of action (Techio et al., 2011).

Negative emotions, particularly anger, activate protest when they are associated to the perception of injustice by the ingroup. That is the frame of diagnosis. But apart from thinking about the current situation, the ingroup also looks at the future and the possibility to change their situation. In this case protest is linked to positive emotions such as hope (Bar-Tal et al., 2007; Páez et al., 2013; Sabucedo and Vilas, 2014).

Since these emotions are generated during intergroup relations and are a response to how political actors interpret them, De Rivera (1992) prefers to talk about emotional climate, rather than emotions. In fact, as Páez et al. (1997) state, emotional climate could be understood as a social representation of the social and political environment.

Once emotional climate has been linked to a determined interpretation of the environment, it is legitimate to consider its influence on certain tendencies of action. This is the argument used by Frijda (1988) to link emotions to different behaviors. In this sense, it is plausible for emotional climate to be relevant in predicting collective actions (Rimé, 2007; Techio et al., 2011). In fact, Páez et al. (2013), found that participants in the Indignados movement had a more negative perception of the emotional climate than non-participants.

It may be concluded from the results of this study that the perception of a negative emotional climate is a necessary condition for collective action. Without a doubt, this is the case most times. But, it is also possible that the protest takes place even in a positive emotional climate. This can happen in those cases where the movement has achieved part of its objectives and has resources to continue advancing. In that situation, positive emotions, usually associated with a better future, activate. This is due to the fact that achievements have empowered participants and they are convinced that carrying on will lead them to success.

Considering all the above, this study performs a comparative design analyzing two relevant collective actions during the political and economic crisis in Spain: one in favor of Catalonian independence and another called Marches for dignity.

The independence movement, until the beginning of the crisis in 2008, had had little influence in Catalonia. However, from that moment on, the movement increased its social support. The claim for independence is mainly based on two arguments: an unfair treatment by Spain and that their lives would be better if they are independent from Spain. Groups and parties that supported independence have governed a large majority of Catalonian political institutions and started to control an important number of organizations and media in Catalonia (Ruiz-Marull, 2016).

The second movement, Marches for dignity, stems from the Indignados movement which arose in Spain in response to the economic and political crisis. At that time, the unemployment rate, over 30%, was the highest in Europe (Eurostat, 2011; Instituto Nacional de Estadística [INE], 2011), and unemployment in 16–25 year olds reached 44% (Instituto Nacional de Estadística [INE], 2011). On the other hand, 50% of the respondents in the barometer of November 2010 (Centro de Investigaciones Sociológicas [CIS], 2010) felt unsatisfied with the performance of their democracy and political leaders. Marches for dignity claims for economic and social rights, especially for those who suffered the consequences of the economic crisis, and for a more egalitarian society. This movement does not have the institutional or media resources the Catalonian independent movement does.

Tens of thousands of people participated in these demonstrations. Moreover, an important amount of the population support both the Independence movement (Centre d‘Estudios d‘Opinion, 2013) and the Indignados movement and the Marches for Dignity (Centro de Investigaciones Sociológicas [CIS], 2011; Garea, 2014).

Taking the aforementioned into account, the following hypotheses are put forward:

(1) The movement of the Marches for dignity constructs a more inclusive identity than the Catalonian independence movement. As a consequence, the causes for the perceived injustice, emotional climate described and the adversary will be different for both movements.
(2) Participants in both mobilizations will have a perception of injustice higher than that of non-demonstrators.
(3) Participants in the Marches for dignity will have a more negative and hostile perception of the emotional climate than the ones in Catalonian independent movement actions and those not participating.
(4) Participants in Catalonian independent movement actions, having more institutional and media resources, and after having achieved certain objectives, will have a more positive outlook on the emotional climate than the participants in the Marches for dignity, and more negative than non-participants.

Two studies were performed. The first one to prove hypothesis number 1, and the second, to prove hypotheses 2, 3, and 4.

## Study 1

A comparative analysis of the manifestos published by both social movements considered in the study: The Catalonian Independence Movement and Marches for dignity, was carried out. This includes an analysis of identified categories in the published manifestos.

### Materials and Methods

The manifestos published by the movements were analyzed: The Catalonian National Assembly (Asamblea Nacional Catalana, 2011, Spanish version) which organizes independence mobilizations, and Marches for Dignity (2016).

#### Procedure

For content analysis, software ATLAS.ti^®^version 7.5 was used and open coding was performed by two expert judges. Through an analysis of words and sentences, they identified concepts and categories based on the references of frames of collective action and emotional climate (Strauss and Corbin, 2002).

### Results

The comparative analysis between both manifestos was structured around three main categories: collective identification, diagnostic framework and emotional climate and injustice causes.

**FIGURE 1:**
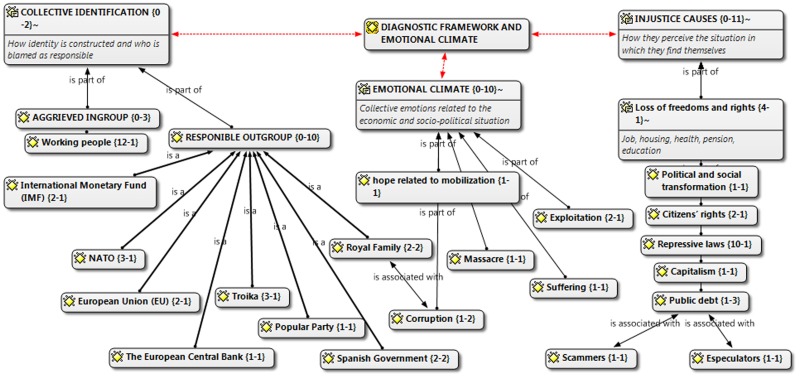
Manifesto marches for dignity.

**Figure 1** shows the results for Marches for dignity. In collective identification, the aggrieved group is the working class (12-1). The values in braces refer to the number of times that subcategory is mentioned in the text {12} and the category where they belong {1}.

In referring to such a wide social category, the Marches for Dignity movement endeavors to make citizens identify with this aggrieved group. As regards those responsible for the situation, the manifesto points toward national (Spanish government, royal family) as well as international (EU, IMF, European Central Bank). institutions. Thus, in this manifesto the crisis and its consequences are interpreted as a struggle between two well-defined social categories: people versus political and economic elite. In the injustice causes category, economic and political grievances appear which show the opposed interests of the aforementioned groups. As a consequence of this, the identified emotional climate is framed in a negative context, exploitation and suffering, among others.

**FIGURE 2:**
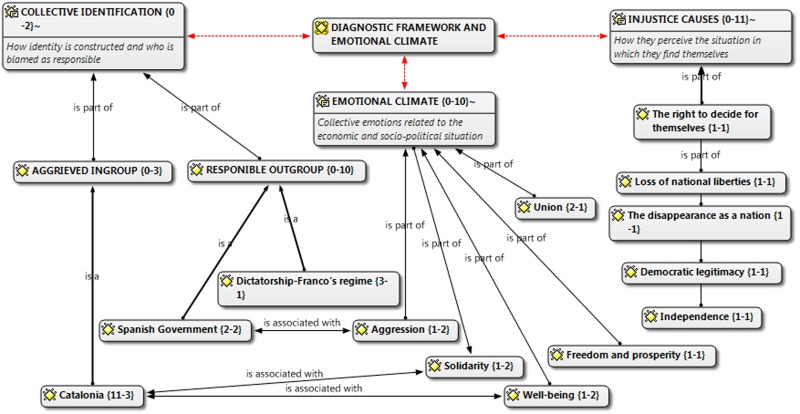
Manifesto for independence.

**Figure 2** shows the category analysis of the manifesto of the Catalonian National Assembly. In this case, the aggrieved group is not a determined social group, as was the case of working people in the Marches for Dignity, but a whole region: Catalonia {11-3}. A region which associates itself with solidarity and well-being. Those responsible for the situation of perceived injustice in Catalonia is The Spanish Government and Franco’s Dictatorship, a regime that ceased to exist 40 years ago. Given that the objectives of the movement is to achieve independence from Spain, the manifesto makes no criticism of the Regional Government of Catalonia’s (in favor of independence) government, nor other international political or economic organisms. In contrast to Marches for Dignity, this manifesto expresses a positive emotional climate. This is, without a doubt, one of the most surprising results and will be analyzed in further detail in Study 2.

**FIGURE 3:**
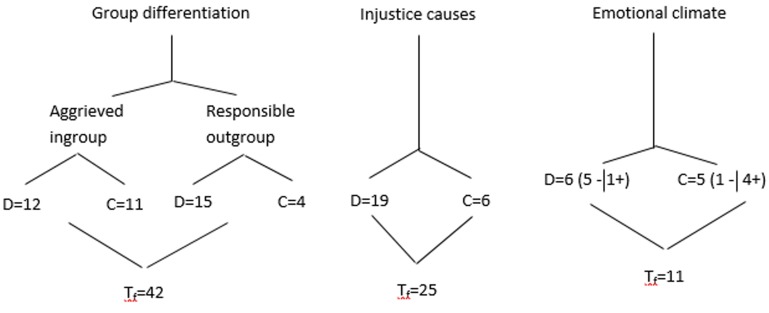
Frequencies of central categories.

Finally, **Figure 3** shows an analysis based on total frequencies of the central categories on which the comparative analysis was structured. The results show the discourse structure is based on ingroup differentiation that each group constructs differently depending on the causes of the injustice. Therefore, there is correspondence between the definition of outgroup responsible and causes for injustice. The frame of diagnosis and emotional climate shows that the manifesto for Marches for dignity (D) uses more negative expressions (D = 5-) compared to the manifesto for the Catalonian National Assembly, which contrarily, uses more positive expressions (C = 4+).

This frequency analysis ratifies the aforementioned comments about the higher number of responsible agents identified in the Marches for Dignity manifesto and its more negative assessment of emotional climate.

## Study 2

Whilst study 1 analyzed the manifestos of the groups that supported collective action, study 2 centered on the participants in the mobilizations. The objective was to know the perception of injustice and emotional climate of participants in the independence movement and the Marches for dignity. Two MANOVA of 2 (Type of demonstration: independence movement, Marches for dignity) × 2 (Type of participant: participant, non-participant) were carried out. In the first MANOVA, dependent variables were perception of injustice, positive emotional climate and negative emotional climate. In the second MANOVA, dependent variables were each of the 9 items on the emotional climate scale.

### Method

#### Sample

The sample was made up of a total of 919 people. For the demonstration conveyed by the Catalonian National Assembly, 376 people were interviewed during the mobilization that took place the 11th of September of 2013 (253 participants and 123 non-participants), participants mean age and sex were 42.5 years and 46.3% respectively. Non-participants were 49.6% women with a mean age of 44.1. There were no significant differences in these two variables. The refusal rate for interviews was 5.8% for participants, and 21.9% among non-participants.

For the demonstration conveyed by the Marches for dignity the 22nd of March of 2014, 543 people were interviewed (278 participants and 265 non-participants), participants mean age and sex were 40.1 years and 48.9% respectively. Non-participants were 53.3% women with a mean age of 39.2. There were no significant differences in these two variables. The refusal rate for interviews were 2.9% for participants, and 16.9% among non-participants.

The present study meets the Ethical principles of psychologists and code of conduct proposed by the American Psychological Association (2002) as well as those ethical regulations made by the Ethical Committee of the University of Santiago de Compostela for Social Science studies with people, that is, fulfilling the requirements of informed consent and data protection (Organic Law 15/1999). Therefore, before the interview was made, the selected participants were informed about the objectives of the study. They were explicitly informed of the voluntariness of their participation, their anonymity, and the confidentiality of their answers, as well as the possibility of stopping their participation at any time during the administration of the questionnaire.

#### Procedure

Participants were selected from within the people who were present in the mobilizations. Thus, avoiding possible bias that may occur in this kind of research when asked about future intentions to participate or past experiences of participation (Conway and Ross, 1984; Ajzen and Sexton, 1999). Non-participants were selected from among those who were walking by or doing other leisure activities at the same time and close to where the demonstration was taking place.

To guarantee all the participants in the demonstrations had the same chances to be interviewed, the procedure designed by Walgrave and Verhulst (2011) was used. The ultimate objective of using this methodology is to avoid the biases that may occur in the selection of the sample. This is an important issue, since in such acts it is common for groups that share the same position to go together or to be together during the event. Therefore, the selection of people cannot be made in a few places, but must be done, randomly, throughout the space that occupies the demonstration.

For following this methodology, the working team is split in two: pointers and interviewers. Each pointer oversees four or five interviewers. The pointer will determine who will be approached by an interviewer. It is important that “select” and “interview” are separate, because interviewers tend to approach people they like or look for those who find them more accessible. The pointers are the ones who decide which demonstrator the interviewer should approach and guarantee the application of the sampling procedure. 20 interviewers and 5 pointers were used in each demonstration.

In the case of non-participants, the same method was used, but on people not taking part in the demonstration. 15 interviewers and 4 pointers were used in each demonstration.

#### Measures

##### Perception of injustice

A scale of 4 items was elaborated which referenced the specific situation that had provoked the mobilization. The answer scale went from 1 to 7, from very unfair to very fair. In the case of the independence movement, one of the items was: “The Spanish government treats Catalonia unfairly.” The alpha value of the scale was 0.72. In the Marches for dignity, one of the items was: “I’m in favor of not paying the debt” The alpha value was 0.75.

##### Emotional climate

The Páez et al. (1997) emotional climate scale was used. The scale has nine items that measure the positive socio-emotional climate (six items) and negative socio-emotional climate (three items). The alpha value of the positive emotional climate subscale was 0.82 and 0.73 for the negative one.

### Results

To make the results clearer, for each MANOVA we refer to the degree of significance [values of *F* and eta squared (η^2^) as size effect index] of the most relevant factors and their interactions. The possible influence on the dependent variables are also indicated. Furthermore, a table with the mean score for the different groups is shown for each MANOVA.

In the first MANOVA of 2 (Type of demonstration: Catalonian independence movement, Marches for dignity) × 2 (Type of participant: participant, non-participant), perception of injustice, positive emotional climate and negative emotional climate were used as dependent variables. Mean scores are shown in **Table 1**.

**Table 1:** Mean scores in perception of injustice and emotional climate (positive and negative) according to type of demonstration (Catalonia Independence, Marches of Dignity) and type of participation (participants, non-participants) and their interaction.

Factors	Type of demonstration		Type of participation		Interaction			
DVs	Independence	Dignity	Participants	Non-participants	Independence		Dignity	
					Participants	Non-participants	Participants	Non-participants
Injustice	5,64	6,11	6,52	5,14	6,38	4,11	6.65	5,58
Positive emotional climate	3,33	2,25	2,87	2,44	3,60	2,75	2,20	2,30
Negative emotional climate	2,58	3,60	3,01	3,43	2,36	3,08	3,60	3,61

With regard to perception of injustice, the two main factors: type de demonstration (*F*(1,935) = 140,13, η^2^ = 0.130) and type of participation (*F*(1,935) = 513,98, η^2^ = 0.355), were significant (*p* < 0.001). The sample for the Independence movement scores lower than that of the Marches for dignity, and participants in demonstrations score higher than non-participants.

The interaction between both factors is also significant (*F*(1,935) = 67,58, *p* < 0.01, η^2^ = 0.067). In this case, it must be highlighted that the difference between participants versus non-participants is substantially greater in the Catalonian independence movement than in the Marches for dignity. This means that there is a greater consensus between participants and non-participants on motives which encourage demonstration in the Marches for Dignity than those in the movement for the independence from Spain. In the latter case, the non-participants in Catalonia show a smaller degree of acceptance of the frame of diagnosis of injustice defended by participants in the demonstration for Catalonian independence.

Considering the positive emotional climate, there is also a significant effect of the factors type of participation (*F*_(1,935)_ = 409,77, *p* < 0.001, η^2^ = 0.305) and type of demonstration (*F*_(1,935)_ = 67.01, *p* < 0.01, η^2^ = 0.067). In this case, the sample from the independence movement obtains the highest mean, and the participants score higher than non-participants.

The analysis also shows a significant effect of the interaction between type of participation and type of mobilization (*F*(1,935) = 109,20, *p* < 0.001, η^2^ = 0.105). Those participating in the Catalonian independence movement are those who have a more positive perception of the emotional climate, while participants in the March for dignity score lower in this variable.

Finally, as regards negative emotional climate, both factors have a significant effect on the dependent variable: type of demonstration *F*(1,935) = 187,27, *p* < 0.001, η^2^ = 0.167), and type of participation (*F*(1,935) = 26,10, *p* < 0.01, η^2^ = 0.027. Those of Marches for dignity and non-participants score highest. The interaction between type of demonstration and type of participation is also significative (*F*(1,935) = 25,19, *p* < 0.01, η^2^ = 0.026), which helps better understand the previous results. That is, the participants in the demonstration for independence are less pessimistic, while the participants and non-participants in the Marches for Dignity are more pessimistic.

To go further into the results of the subscales, a second MANOVA of 2 (Type of demonstration: Catalonian independence movement, Marches for dignity) × 2 (Type of participant: participant, non-participant) was carried out. In this case the dependent variables were each of the nine items on the emotional climate scale. Mean scores for the different groups are shown in **Table 2**.

**Table 2:** Mean scores in the items of emotional climate scale (positive and negative) according to type of demonstration (Independence, Dignity) and type of participation (participants, non-participants) and their interaction.

Factors	Type of demonstration		Type of participation		Interaction			
DVs	Independence	Dignity	Participants	Non-participants	Independence		Dignity	
					Participants	Non-participants	Participants	Non-participants
Good general affective climate	3,39	2,05	2,69	2,45	3,56	3,02	1,89	1,20
Emotional atmosphere of hope	3,61	2,14	3,90	3,02	3,90	3,02	2,02	2,25
Emotional atmosphere of solidarity	3,89	3,62	2,93	2,49	4,24	3,16	3,69	3,55
Emotional atmosphere of confidence	2,39	1,39	1,92	1,63	2,66	1,85	1,23	1,54
Emotional atmosphere of fear	2,59	3,43	2,92	3,31	2,41	2,98	3,41	3,46
Emotional atmosphere of hostility	2,47	3,57	2,92	3,39	2,24	2,94	3,56	3,58
Emotional atmosphere of sadness	2,67	3,86	3,18	3,63	2,42	3,18	3,89	3,82
Emotional atmosphere of happiness	2,98	1,67	2,49	1,83	3,33	2,26	1,71	1,64
Emotional atmosphere of tranquility	3,78	2,59	3,28	2,80	4,04	3,26	2,58	2,59

With respect to positive emotions, the two main factors (type of demonstration and type of participation) have significant effects on the dependent variables: good affective climate, hope, confidence, happiness and being at ease to speak. In all of them, the samples from Marches for dignity show a significantly worse perception than those from the Catalonian independence movement. The only exception is seen in the factor type of demonstration. The variable solidarity does not present significant differences (*F*(1,910) = 1,32, η^2^ = 0.001).

The interaction between factors is significant in all variables (*p* < 0.001): good affective climate, (*F*(1,910) = 31,12, η^2^ = 0.033), hope (*F*(1,910) = 53.45, η^2^ = 0.055), solidarity (*F*(1,910) = 45.82, η^2^ = 0.048), confidence (*F*(1,910) = 80.65, η^2^ = 0.081), happiness (*F*(1,910) = 51.08, η^2^ = 0.053) and being at ease to speak (*F*(1,910) = 21,00, η^2^ = 0.023). As can be seen in **Table 2**, participants in the Catalonian independence movement and in Marches for Dignity have a completely different perception of emotional climate. The former score highest in all those variables while the latter obtain the lowest scores in three of them (hope, confidence, and tranquility). It is important to underline that the participants in the independence movement score higher in solidarity, but the non-participants score the lowest of the four groups.

As regards negative emotions, the results complement those above. Both factors have significant effects on these dependent variables: fear, hostility, and sadness. Marches for Dignity and non-participants score the highest. However, comprehending these results is easier by analyzing interactions. Interaction was significant for negative emotions: fear (*F*(1,910) = 8,80, *p* < 0.001, η^2^ = 0.010), hostility (*F*(1,910) = 14.61, η^2^ = 0.016), sadness (*F*(1,910) = 25,69, *p* <0.001, η^2^ = 0.027). As can be observed in **Table 2**, the group of participants in the independence movement is the one which perceives, in significant measure, that the atmosphere is not as negative in each of these three emotions. The non-participants in the Catalonian independence movement have a significantly more negative perception than participants. Participants and non-participants in the Marches for Dignity perceive emotional climate more negatively.

## Discussion

The first study in this manuscript shows the capacity of the movements to create meanings and interpretations of reality. This stood out when identifying the victimized group and those responsible for the situation. The crisis affected the whole of Spain and the politics of economic cuts was undertaken by both the State and Autonomic governments following the policies approved in the European Union Commission. Despite this, in Catalonia the nationalist message, supported by the Catalonian government (Orriols, 2012) took root, pointing out the Spanish government as mainly responsible for the crisis.

Obviously, this independence discourse did not appear overnight. It had been present for a long time in a small part of Catalonian society. The economic and political crisis generated anger toward the current system and shed doubt on its causes and solutions. The nationalist offer gave a simple and attractive explanation about the causes and an easy cure to recover well-being: it is Spain’s fault, if we become independent, we will live better. Together with this, they appealed to the epic achievement of liberty and dignity for the ingroup. Thus, in the manifesto, more positive references than negative ones are seen.

The March for dignity movement constructed a completely different interpretation. The subject of injustice is not the nation, but specific people who suffer cuts in their economic and social rights and liberties. It is a more inclusive identity that does not differentiate people because of where they are from, but due to the conditions in which they live.

The inclusive character of the ingroup is also applied to the other responsible party. It is not a state or government, but an economic and political system. That is why the European Union, the International Monetary Fund or the Troika get signaled out. They are the executors of policies that make the population suffer. That attribution of responsibilities coincided with the disaffection citizens had been showing toward the political establishment, regardless of whether right or left wing. This movement, like Indignados, expressed the general uneasiness toward the system. The little real and institutional power of this movement makes its manifesto use more negative than positive emotional references.

The second study, performed with participants in the mobilizations called for by both movements, goes deeper into perceived injustice and emotional climate.

As regards the ingroup’s perception of the situation, the results support our hypothesis. Participants in both demonstrations feel more unfairly treated than non-participants. Apart from this, there are two results of interest in relation to the Independence movement. Firstly, the group with lesser perception of injustice is that of the non-participants in that movement. Secondly, despite there being significant differences among all the groups, the participants and non-participants in the independence movement show the greatest divergence in this dimension. This means that non-participants do not share the same diagnosis than participants as regards Catalonia being mistreated by Spain. The lack of a widespread consensus on this issue may cause society to fracture due to the identity, economic and social factors involved.

Finally, the relation between perceived emotional climate and collective action was analyzed. Previous studies had shown that a negative emotional climate was associated with collective action. In our study, this is only the case in the Marches for dignity. In the case of the participants of the Independence movement we find, as we had foreseen, that their perception of emotional climate is better than that of the Marches for dignity. However, contrary to what we had suggested, their perception of emotional climate is also more positive than that of the non-participants. This means that participants in two collective actions demanding change, the Catalonian independence movement and the Marches for dignity, are on opposite sides of the emotional climate continuum.

In the case of the Catalonian independence movement, this has the support of an important number of citizens, but especially from the Catalonian Government which implies more resources and the means to spread the movement’s claims. Furthermore, its discourse reinforces the social identity of the group which contributes to a better self-concept of those more identified with it Tajfel and Turner (1979). Despite considering Catalonia is being unfairly treated, this leads to a positively perceived emotional climate.

The Marches for dignity also had significant social support. However, they did not count on the support of the institutions or governments. They did not have the necessary resources to believe the situation could change in the short-term, either. Therefore, as with the majority of social movements, their perception of the emotional climate was negative.

One of the limitations of this study is that we only take into account if participants have the feeling of injustice without asking them about the reason behind that perception of unfairness (it could be for cultural or economic reasons, which in this case we cannot know). However, the main objective of this work wasn’t to know the reasons that lead to the injustice frame, but to find out if that feeling exists.

Another limitation is that we analyzed a movement with the characteristics of Catalonian independence. To generalize those results, other similar movements, with the same status and power in relation to the outgroup, should be considered. Also, this work has not taken other important variables into account to explain collective action, such as identification, efficacy or moral obligation (Vilas and Sabucedo, 2012). If we want to set up an integrative model of political actions, those variables would be needed. But at the same time, our study shows that emotional climate has to be an important part of that model.

## Conclusion

Social and political situations of crisis are usually associated with the appearance of citizen mobilizations that demand changes in the system. Nevertheless, not all the collective actions that may arise adopt the same kind of solutions. In the cycle of protest which started in Spain in 2008 we find, among others, two movements. One, for Catalonian independence and the other, the Marches for dignity, which have different answers to the crisis.

For collective action to take place, it is necessary to have a frame of diagnosis which defines a situation as unjust and identifies the aggrieved group as well as the adversary. This work shows how, in the same political and economic crisis, the construction of these identities can be different, depending on its lower or higher inclusiveness. This proves the influence of context and the mobilizing offers in the type and objectives of collective action (van Stekelenburg et al., 2009; Gómez-Román and Sabucedo, 2014). The relevance of context is brought to light in the assessment of emotional climate. The achievement of certain objectives and the reach of the movement seem to have an impact on a positive emotional climate, even though one assumes the current situation is unjust. This way, the comparative design used in this work brings a new perspective to the relation among injustice, emotional climate, and collective action.

## Author Contributions

This has been a work made by a team. All the authors, have done substantial contributions to the conception and design of the work; also in the acquisition, analysis, and interpretation of data for the work. All have taken part in the process of drafting and revising the work and all of them agree that in the current state is valid to be submitted. They also agree questions related to the accuracy and integrity of the work are appropriately investigated and solved.

## Conflict of Interest Statement

The authors declare that the research was conducted in the absence of any commercial or financial relationships that could be construed as a potential conflict of interest.

## References

[B1] Ajzen I. Sexton J. (1999). “Depth of processing, belief congruence, and attitude behavior correspondence,” in *The Dual Process Theories in Social Psychology*, eds Chaiken S. Tope Y. (New York, NY: Guilford), 117–138.

[B2] American Psychological Association (2002). *Publication Manual of the American Psychological Association*, 6th Edn. Washington, DC: American Psychological Association.

[B3] Asamblea Nacional Catalana, [ANC] (2011). *Manifest de Suport.* Available at: http://assemblea.cat/?q=manifest_de_suport/

[B4] Bar-Tal B. Halperin E. de Rivera J. (2007). Collective emotions in conflict situations: societal implications. *J. Soc. Issues* 63 441–460. 10.1111/j.1540-4560.2007.00518.x

[B5] Bergstrand K. (2014). The mobilizing power of grievances: applying loss aversion and omission bias to social movements. *Mobilization* 19 123–142.

[B6] Centre d‘Estudios d‘Opinion (2013). *REO n° 733, 22 de Noviembre de 2013.* Available at: http://ceo.gencat.cat/ceop/AppJava/pages/estudis/categories/fitxaEstudi.html?colId=4688&lastTitle=Bar%F2metre+d%27Opini%F3+Pol%EDtica+%28BOP%29.+3a+onada+2013 [accessed July 27, 2017].

[B7] Centro de Investigaciones Sociológicas [CIS] (2010). *Barómetro del Mes de Noviembre.* Available at: http://www.cis.es/cis/opencms/ES/11_barometros/indicadores.html [accessed March 14, 2012].

[B8] Centro de Investigaciones Sociológicas [CIS] (2011). *Barómetro del Mes de Junio. Estudio n° 2905.* Available at: http://www.cis.es/cis/opencms/ES/11_barometros/indicadores.html [accessed July 27, 2017].

[B9] Conway M. Ross M. (1984). Getting what you want by revising what you had. *J. Pers. Soc. Psychol.* 47 738–748. 10.1037/0022-3514.47.4.738

[B10] Cristancho C. (2015). “A tale of two crises: contentious responses to anti-austerity policy in Spain,” in *Austerity and Protest: Popular Contention in Times of Crisis*, eds Giugni M. Grasso M. (Abingdon: Routledge), 193–216.

[B11] De Rivera J. H. (1992). “Emotional climate: social structure and emotional dynamics,” in *International Review of Studies on Emotion*, ed. Strongman K. T. (New York, NY: John Wiley & Sons), 197–218.

[B12] Eurostat (2011). *Euro-Indicators.* Available at: http://ec.europa.eu/eurostat [accessed February 04, 2015].

[B13] Eyerman R. Jamison A. (1991). *Social Movements: A Cognitive Approach.* University Park, PA: The Pennsylvania State University Press.

[B14] Frijda N. H. (1988). The laws of emotion. *Am. Psychol.* 43 349–358. 10.1037/0003-066X.43.5.349 3389582

[B15] Gamson W. A. (1992). *Talking Politics.* Cambridge: Cambridge University Press.

[B16] Garea F. (2014). *Podemos Supera a PSOE y PP y Rompe el Tablero Electoral.* Available at: https://politica.elpais.com/politica/2014/11/01/actualidad/1414865510_731502.html [accessed November 2, 2014].

[B17] Goffman E. (1974). *Frame Analysis.* Cambridge: Harvard University Press.

[B18] Gómez-Román C. Sabucedo J. M. (2014). The importance of political context: motives to participate in a protest before and after the labor reform in Spain. *Int. Sociol.* 29 546–564. 10.1177/0268580914549861

[B19] Instituto Nacional de Estadística [INE] (2011). *Encuesta de Población Activa.* Available at: http://www.ine.es/jaxiBD/tabla.do?per=12&type=db&divi=EPA&idtab=756 [accessed February 04, 2015].

[B20] Klandermans B. (2004). “The demand and supply of participation: social-psychological correlates of participation in social movements,” in *The Blackwell Companion to Social Movements*, eds Snow D. A. Soule S. A. Kriesi H. (Oxford: Blackwell), 360–379.

[B21] Lazarus R. S. (1984). On the primacy of cognition. *Am. Psychol.* 39 124–129. 10.1037/0003-066X.39.2.124

[B22] Marches for Dignity. (2016). *La Lucha es el Único Camino.* Available at: https://marchasdeladignidadmadrid.wordpress.com/2016/03/01/la-lucha-es-el-unico-camino [accessed March 01, 2017].

[B23] Ministry for Home Affairs of the Spanish Government (2014). *Anuario Estadístico del Ministerio del Interior.* Madrid: Ministerio del Interior.

[B24] Orriols L. (2012). *El Relato Nacionalista de la Crisis.* Available at: https://elpais.com/ccaa/2012/11/21/catalunya/1353521519_788266.html [accessed November 21, 2012].

[B25] Páez D. Javaloy F. Wlodarczyk A. Espelt E. Rimé B. (2013). El movimiento 15-M: sus acciones como rituales, compartir social, creencias, valores y emociones. *Rev. Psicol. Soc.* 28 19–33. 10.1174/021347413804756078

[B26] Páez D. Ruiz J. I. Gailly O. Kornblit A. L. Wiesenfeld E. Vidal C. M. (1997). Clima emocional: su concepto y medición mediante una investigación transcultural. *Rev. Psicol. Soc.* 12 79–98. 10.1174/021347497320892045

[B27] Rimé B. (2007). The social sharing of emotion as an interface between individual and collective processes in the construction of emotional climates. *J. Soc. Issues* 63 307–322. 10.1111/j.1540-4560.2007.00510.x

[B28] Ruiz-Marull D. (2016). *Breve Historia de un Proceso Independentista. La Vanguardia.* Available at: http://www.lavanguardia.com/politica/20161230/412969825085/ceo-historia-proceso-catalunya-independencia.html [accessed December 30, 2016].

[B29] Sabucedo J. M. Durán M. Alzate M. (2010). Identidad colectiva movilizada. *Rev. Psicol. Soc.* 25 189–201. 10.1174/021347410791063822

[B30] Sabucedo J. M. Grossi J. Fernández C. (1998). “Los movimientos sociales y la creación de un sentido común alternativo,” in *Los Movimientos Sociales*, eds Ibarra P. Tejerina B. (Madrid: Ed. Trotta), 165–180.

[B31] Sabucedo J. M. Vilas X. (2014). Anger and positive emotions in political protest. *Univ. Psychol.* 13 829–838. 10.11144/Javeriana.UPSY13-3.apep

[B32] Simon B. Klandermans B. (2001). Politicized collective identity: a social psychological analysis. *Am. Psychol.* 56 319–331. 10.1037/0003-066X.56.4.319 11330229

[B33] Smith E. R. (1993). “Social identity and social emotions: toward new conceptualizations of prejudice,” in *Affect, Cognition, and Stereotyping: Interactive Processes Ingroup Perception*, eds Mackie D. M. Hamilton D. L. (San Diego, CA: Academic Press), 297–315.

[B34] Strauss A. L. Corbin J. (2002). *Bases de la Investigación Cualitativa: Técnicas y Procedimientos Para Desarrollar la Teoría Fundamentada.* Medellín: Editorial Universidad de Antioquia.

[B35] Tajfel H. Turner J. C. (1979). “An integrative theory of intergroup conflict,” in *The Social Psychology of Intergroup Relations*, eds Austin W. G. Worchel S. (Monterey, CA: Brooks/Cole), 33–47.

[B36] Tarrow S. (1991). *Struggle, Politics, and Reform: Collective Action, Social Movements and Cycles of Protest.* Ithaca, NY: Cornell University.

[B37] Tarrow S. (1993). Cycles of collective action: between moments of madness and the repertoire of contention. *Soc. Sci. Hist.* 17 281–307. 10.2307/1171283

[B38] Techio E. Zubieta E. Páez D. de Rivera J. Rimé B. Kanyangara P. (2011). “Clima emocional y violencia colectiva: El estado de la cuestión y los instrumentos de medición,” in *Superando la Violencia Colectiva y Construyendo Cultura de Paz*, eds Páez D. Martin Beristain C. González J. L. Basabe N. de Rivera J. (Madrid: Fundamentos), 103–148.

[B39] Turner R. H. Killian L. M. (1987). *Collective Behavior*, 3rd Edn. Englewood Cliffs, NJ: Prentice-Hall, Inc.

[B40] van Stekelenburg J. Klandermans B. van Dijk W. W. (2009). Context matters: explaining how and why mobilizing context influences motivational dynamics. *J. Soc. Issues* 65 815–838. 10.1111/j.1540-4560.2009.01626.x

[B41] van Stekelenburg J. Klandermans B. van Dijk W. (2011). Combining motivations and emotion: the motivational dynamics of protest participation. *Rev. Psicol. Soc.* 26 91–104. 10.1174/021347411794078426

[B42] van Stekelenburg J. Klandermans K. (2014). Fitting demand and supply: how identification brings appeals and motives together. *Soc. Mov. Stud.* 13 179–203. 10.1080/14742837.2013.843448

[B43] van Zomeren M. Postmes T. Spears R. (2008). Toward an integrative social identity model of collective action: a quantitative research synthesis of three socio-psychological perspectives. *Psychol. Bull.* 134 353–372. 10.1037/0033-2909.134.4.504 18605818

[B44] Vilas X. Sabucedo J.-M. (2012). Moral obligation: a forgotten dimension in the analysis of collective action. *Rev. Psicol. Soc.* 27 369–375. 10.1174/021347412802845577

[B45] Walgrave S. Verhulst J. (2011). Selection and response bias in protest surveys. *Mobilization* 16 203–222.

